# *Sipha maydis* sensitivity to defences of *Lolium multiflorum* and its endophytic fungus *Epichloë* occultans

**DOI:** 10.7717/peerj.8257

**Published:** 2019-12-18

**Authors:** Daniel A. Bastías, Maria Alejandra Martínez-Ghersa, Jonathan A. Newman, Stuart D. Card, Wade J. Mace, Pedro E. Gundel

**Affiliations:** 1 IFEVA, Consejo Nacional de Investigaciones Científicas y Técnicas, Universidad de Buenos Aires, Buenos Aires, Argentina; 2 Forage Science, AgResearch Limited, Grasslands Research Centre, Palmerston North, New Zealand; 3 Department of Biology, Wilfrid Laurier University, Waterloo, Ontario, Canada

**Keywords:** Alkaloids, Beneficial microorganisms, Endophyte symbiosis, *Epichloë* fungalendophytes, Plant defences, Salicylic acid, Plant-herbivore interaction

## Abstract

**Background:**

Plants possess a sophisticated immune system to defend from herbivores. These defence responses are regulated by plant hormones including salicylic acid (SA) and jasmonic acid (JA). Sometimes, plant defences can be complemented by the presence of symbiotic microorganisms. A remarkable example of this are grasses establishing symbiotic associations with *Epichloë* fungal endophytes. We studied the level of resistance provided by the grass’ defence hormones, and that provided by *Epichloë* fungal endophytes, against an introduced herbivore aphid. These fungi protect their hosts against herbivores by producing bioactive alkaloids. We hypothesized that either the presence of fungal endophytes or the induction of the plant salicylic acid (SA) defence pathway would enhance the level of resistance of the grass to the aphid.

**Methods:**

*Lolium multiflorum* plants, with and without the fungal endophyte *Epichloë occultans*, were subjected to an exogenous application of SA followed by a challenge with the aphid, *Sipha maydis*.

**Results:**

Our results indicate that neither the presence of *E. occultans* nor the induction of the plant’s SA pathway regulate *S. maydis* populations. However, endophyte-symbiotic plants may have been more tolerant to the aphid feeding because these plants produced more aboveground biomass. We suggest that this insect insensitivity could be explained by a combination between the ineffectiveness of the specific alkaloids produced by *E. occultans* in controlling *S. maydis* aphids and the capacity of this herbivore to deal with hormone-dependent defences of *L. multiflorum*.

## Introduction

To defend from herbivore attacks, plants harbour a sophisticated immune system in which hormone pathways including salicylic acid (SA) and jasmonic acid (JA) mediate defence responses. These hormone pathways are known to be differentially involved in the response to distinct natural enemies. While the plant SA-dependent defence pathway is generally induced by sap-sucking insect herbivores and biotrophic pathogens, the JA-dependent defence pathway acts in response to chewing insect herbivores and necrotrophic pathogens (Thaler, Humphrey & Whiteman, 2012; Ballaré, 2014). In some cases, the plant immune system can be complemented by hormone-independent mechanisms of defences. A particularly striking example of this is provided by grasses establishing symbiotic associations with *Epichloë* fungal endophytes (Clay, 1988; Bastias et al., 2017a; Bastías et al., 2017b). These fungi protect their host grasses against herbivores by the production of bioactive alkaloids (Panaccione, Beaulieu & Cook, 2014).

Some grasses establish symbiotic associations with asexual *Epichloë* fungal species that are strictly vertically-transmitted. These grass-*Epichloë* endophyte associations are usually mutualistic, since plants provide the fungus a place to live (the fungus is an obligate symbiont) and the fungus provides the grass with beneficial traits such as anti-herbivore defences (Gundel, Rudgers & Ghersa, 2011; Saikkonen, Saari & Helander, 2010; Schardl et al., 2013a; Schardl et al., 2013b). *Epichloë* fungal endophyte species can collectively synthesize a vast number of secondary metabolites and this diversity of bioactive compounds provides their host grasses defences against a range of herbivore species (Saikkonen, Saari & Helander, 2010; Schardl et al., 2013a; Schardl et al., 2013b). Four classes of *Epichloë*-derived alkaloids have been well-studied: pyrrolizidines (e.g., lolines), peramine, indole-diterpenes (e.g., lolitrem B, terpendoles), and ergot alkaloids (e.g., ergovaline) (Panaccione, Beaulieu & Cook, 2014; Saikkonen, Gundel & Helander, 2013; Schardl et al., 2013a; Schardl et al., 2013b). The alkaloid profile produced depends on the fungal species/strain, with some species/strains producing only one type of alkaloid (Schardl et al., 2013a; Schardl et al., 2013b). Moreover, the effectiveness of a given alkaloid type depends on its concentration and the herbivore species (Fuchs et al., 2017b; Bastias et al., 2017a; Bastías et al., 2017b; Bultman et al., 2018). Alkaloid production is dependent on the fungal biomass, herbivory level, plant ontogenic stage and nutritional status, plant tissue type, and some abiotic conditions (e.g., temperature, CO_2_ levels) (Justus, Witte & Hartmann, 1997; Hunt et al., 2005; Rasmussen et al., 2007; Ryan et al., 2014; Fuchs et al., 2017a; Fuchs et al., 2017b; Gundel et al., 2018; Bultman et al., 2018).

The presence of biotrophic pathogens within plants is usually controlled by SA-dependent responses (Thaler, Humphrey & Whiteman, 2012). Recent research shows that the same pathway can also regulate the interaction with beneficial symbionts. For instance, the plant induction of the SA pathway inhibits the development and establishment of rhizobacterial and mycorrhizal symbionts in plant tissues (Cao et al., 2017; Bedini et al., 2018). Moreover, plant SA immune responses can also affect the activities performed by plant beneficial symbionts. For example, alkaloid production and nitrogen fixation performed by beneficial symbionts is reduced by the induction of the SA pathway in grasses, legumes, and ferns (Hayat et al., 2010; Bastías et al., 2018a; De Vries et al., 2018). Independently of the control exerted by plants on their beneficial symbionts, these symbionts can, in turn, modulate the SA pathway (Johnson et al., 2003; Stacey et al., 2006; Navarro-Meléndez & Heil, 2014; Dupont et al., 2015; Moreira, Abdala-Roberts & Castagneyrol, 2018; Ramos et al., 2018). For instance, genes encoding proteins pertaining to SA biosynthesis and signalling were downregulated by the presence of the endophyte *E. festucae* Fl1 in *Lolium perenne* plants (Dupont et al., 2015). The symbiont’s suppression of SA-dependent responses might be a mechanism used by these microorganisms to facilitate growth into plant tissues (Jung et al., 2012; Bastías et al., 2018a).

Here, we studied the level of resistance mediated by plant hormones and provided by an *Epichloë* endophyte fungus against an introduced aphid species in grasses. We hypothesized that either the presence of fungal endophytes or the induction of the plant SA pathway would enhance the level of resistance of the grass against the aphid. We subjected Italian ryegrass plants (*Lolium multiflorum*), symbiotic and non-symbiotic with the endophyte *Epichloë occultans* (Moon et al., 2000), to an exogenous SA application followed by a challenge with the hedgehog grain aphid (*Sipha maydis*). This aphid species is native to Eurasia, and individuals feed on cereals and other grasses (Skvarla et al., 2017). In Argentina, where this work was conducted, *S. maydis* was first discovered in 2002 and has since spread throughout temperate grasslands and cropping regions of the country (Corrales et al., 2007). Corrales et al. (2007) reported for this aphid species, average densities from 18 to 98 individuals per plant depending of the season and the geographic location. In temperate grasslands, the first populations were found in 2003 in the south-east of the Buenos Aires province (34°55′S, 57°57′W) (Corrales et al., 2007). In these grasslands, *Sipha maydis* is commonly found feeding on *L. multiflorum* plants with populations reaching sizes of around 250 individuals per plant (Chaneton & Omacini, 2007). The ryegrass *L. multiflorum* is a naturalised and abundant species in the Argentinian temperate grasslands. This species is native to the European Mediterranean region and was introduced in Argentina more than a century ago (Uchitel, Omacini & Chaneton, 2011). While many different alkaloids are produced across the genus *Epichloë*, *E. occultans* produces only loline alkaloids (i.e., N-formylloline (NFL) and N-acetylnorloline (NANL)) (Sugawara et al., 2006; Moore et al., 2015; Bastias et al., 2017a; Bastías et al., 2017b). It has been shown that both the presence of *E. occultans* (Omacini et al., 2001; Miranda, Marina & Chaneton, 2011; Gundel et al., 2012; Ueno et al., 2015; Bastias et al., 2017a; Bastías et al., 2017b), and the loline alkaloids produced by this and other endophyte species provide the plant with protection from aphids (Johnson et al., 1985; Eichenseer, Dahlman & Bush, 1991; Wilkinson et al., 2000; Panaccione, Beaulieu & Cook, 2014).

We predicted that the presence of *Epichloë* fungal endophytes within their host plants would enhance the level of plant resistance against the aphid and consequently reduce the aphid’s performance (i.e., aphid individual metabolic rates and population sizes). In addition, the stimulation of the SA-dependent defence response, by the exogenous application of the hormone, would increase the resistance in non-symbiotic plants, affecting negatively the aphid performance. However, we expected that the SA treatment would affect the endophyte-conferred plant resistance against aphids. Specifically, since *Epichloë* fungal endophytes are biotrophic microorganisms, the exogenously applied SA would impair the *Epichloë*, subsequently reducing its alkaloid production, and thus decreasing the resistance level and consequently, increasing the metabolic rates and population sizes of the aphids.

## Material and Methods

### Plant and aphid stocks

We worked with the annual grass plants of *Lolium multiflorum* both symbiotic (E+) and non-symbiotic (E-) with its common fungal endophyte *E. occultans* (Moon et al., 2000). More than a decade ago, seeds of *L. multiflorum* with high percentages of endophyte infection were hand-collected from a naturalised population in the Pampean grassland (Argentina) (36°00′S, 61°5′W) (Gundel et al., 2009). Immediately after collection, a proportion of these seeds were treated with the systemic fungicide Triadimenol (150 g kg^−1^; Baytan®) in order to obtain endophyte-free individuals. Since then, plants of these two biotypes (i.e., E+ and E-) have been cultivated annually in a common garden (recall that *E. occultans* is strictly vertically transmitted), multiplying fresh seeds for experimentation [IFEVA - CONICET, Universidad de Buenos Aires, Argentina (34°35′S, 58°28′W)]. Genetic segregation between plant biotypes has been prevented by allowing individual plants to freely exchange pollen during flowering (Gundel et al., 2012). Each late spring–early summer, ripe seeds produced by each plant biotype are harvested and evaluated for endophyte presence; after confirming the level of endophytes in each biotype, the seeds are stored in a 4 °C freezer. The endophyte detection is carried out by looking for fungal hypha in stained individual seeds following the “seed squash technique” (Bacon & White, 1994; Card et al., 2011). For this, 100 seeds from each seed lot (E+ and E-), are incubated in NaOH (5%), stained with rose bengal and examined under a light microscope at 40X power. The seeds produced in 2014 were examined for endophyte infection frequency (E+: 99%, and E-: 1%; *n* = 100 each) and stored in cold and dry conditions until use (2015).

In early-spring 2015, individual aphids *S. maydis* (Passerini) were collected from the local extant vegetation dominated by cereals and wild grasses. Starting with approximately 150 apterous adults, an aphid population was established within a growth chamber [21 °C (±1) constant, radiation 150 µmol m^−2^ s^−1^, and photoperiod L16:D8 h] on wheat plants (Var. Cronox; Don Mario). Wheat plants were replaced periodically to provide fresh material for the aphid population. After 6 weeks, the aphid population was large enough to provide the required number of individual adult aphids for the experiment (see next section).

### Experimental description

In 2015 during the normal growing season for *L. multiflorum* (autumn-winter-spring), 50 E+ and 50 E- plants were grown in 1.5 L pots, filled with a mix of soil, sand, and peat in equal proportions. Plants were watered to field capacity, as needed, to avoid water deficits. In early-spring, 28 E+ and 28 E- healthy plants were selected and transferred to a growth chamber with the same environmental conditions as described earlier. At that time, the plants averaged 48 tillers (range: 25–73) and were starting the reproductive stage (spike appearance). After careful examination to ensure there were no invertebrates present on the *L. multiflorum* plants, each plant was individually enclosed within a white cotton voile fabric bag supported by a tubular plastic net. Before the application of the hormone treatment (see below), the plants were acclimated to the growth chamber conditions for one week.

The experiment comprised a 2 × 2 full factorial design, with endophyte (E+, E-) and salicylic acid (SA+, SA-) as the experimental treatments. Fourteen plants from each biotype were sprayed with 10 ml of SA solution (0.5 mM; Biopack®, Argentina). The same procedure was done on the other 14 E+ and 14 E- plants but sprayed with 10 ml of distilled water. Three days later, each plant was challenged with 5 apterous adult aphids. This number of aphids as starting population was chosen based on field observations, where the colonization of *L. multiflorum* plants by *S. maydis* aphids is usually carried out by only few individuals (Chaneton & Omacini, 2007). The 3-days period between the application of SA and the aphid challenge was previously identified as enough time for the plants to develop a defence response prior to contact with aphids (Bastías et al., 2018a).

The aphid populations on the *L. multiflorum* plants developed over the next 24 days. We counted the number of insects on each plant (aphid population size) at days 7 and 14 (corresponding to days 10 and 17 since the SA application, respectively). At day 24 (27th since SA application), a group of approximately 35 randomly chosen aphids were sampled from a subset of 5 individual plants per treatment to measure the mass-specific standard metabolic rate (SMR) by means of open-flow respirometry.

Two serial samples of plant tissues were taken to measure the physiological concentrations of plant defence hormones and fungal alkaloids. The tissues were sampled from a subset of 8 plants per treatment, selected at random. The first sample was taken on day 3, just prior to the introduction of the aphids. Two leaf-blades were excised from one tiller per plant just before the aphid challenge (day 3 post SA application) to evaluate the concentrations of SA and JA hormones. Since the recognized role of JA-signalling pathway responses in plant defences (Thaler, Humphrey & Whiteman, 2012), JA concentration levels were also measured in response to the endophyte presence and the SA treatment. The second harvest comprised the removal of one tiller per E+ plant 7 days after the aphid challenge (10 days post SA application). We used the pseudostem from this tiller to evaluate the concentration of loline alkaloids [note that the fungal endophyte *E. occultans* only produces this type of alkaloid (Bastias et al., 2017a; Bastías et al., 2017b)]. We selected tillers in visibly good conditions but with symptoms of aphids feeding activities. Even though E- plants are incapable of producing loline alkaloids, E- were subjected to the same sampling procedure as E+ plants to avoid any manipulation-dependent effects (Cahill Jr, Castelli & Casper, 2002). We estimated that the total tissue removed for hormone and alkaloid assessments represented around 1% of the aboveground plant biomass. At day 27 post SA application, the total aboveground biomass of the plants was harvested, and immediately dried in an oven (2 d at 60 °C) to evaluate the individual plant dry weight (Analytical scale, ± 0.01 g, Mettler Toledo).

### Quantification of SA and JA hormones

Starting from freeze-dried and ground leaf material, subsamples of 50–100 mg were extracted with 100% Acetonitrile containing 100 ng of d6-SA and d5-JA as internal standards. The extracts were dried, derivatized with N-Methyl-N-(trimethylsilyl)trifluoroacetamide, and injected into an Agilent DB-5MS column (30 m, 0.25 mm inner diameter, 0.25 µm film thickness with a 10 m guard column). The column effluent was added into the ion source of a Scion TQ GC-MS/MS (Bruker Daltonics Inc.). The mass spectrometer was operated in positive ionization mode with multiple reactions monitoring (MRM) as previously described in Bastías et al. (2018a). SA and JA hormones were quantified relative to the peak area of their corresponding internal standards.

### Quantification of loline alkaloids

Lolines were extracted from 50 mg of freeze-dried, ground plant samples using a solution of 40% methanol/5% ammonia and 1,2-dichloroethane containing 54.8 ng mL^−1^ 4-phenylmorpholine as internal standard. Plant extracts were centrifugated, and supernatants transferred to glass GC vials via a 20 µm filter for analysis. The analysis was conducted using a GC flame ionization detector (GC2010Plus, Shimadzu Corporation, Japan) and separation was achieved on a ZB-5 capillary column (30 m × 0.32 mm × 0.25 µm film). More information can be found in Bastías et al. (2018a). The detection limit was 25 µg g^−1^ DW.

### Measurements of standard metabolic rate (SMR)

The SMR quantifies the energy budget required for insects to maintain homeostasis, thus this variable represents a measure of the general physiological status of insects (Nespolo, Roff & Fairbairn, 2008). Aphid maximum annual growth rates (r_m_) are negatively correlated with SMR. Aphids with high metabolic rates (i.e., high levels of maintenance costs) might have less energy available for reproduction thus negatively impacting their population sizes (Castañeda, Figueroa & Nespolo, 2010). The SMR was measured by the production of CO_2_ (VCO_2_) of aphid groups placed within of an open-flow respirometry system (LI-6400; Li-Cor, Lincoln, USA). We estimated the mass-specific SMR that is, the amount of CO_2_ produced per mass unit of aphid per hour (i.e., µL of VCO_2_ per mg of aphid per hour). Aphids were obtained from a subset of 5 randomly chosen plants per treatment. From each of these plants, 35 adult and non-winged aphids were carefully removed and placed in Eppendorf tubes. We considered each group of aphids from one individual plant to be a replicate (i.e., 5 replicates per treatment). All the aphid groups were weighted (±0.01 mg, analytical balance, Mettler Toledo), and kept for one hour without food before the metabolic measurements. The VCO_2_ of each aphid group was registered every second during a period of 10 min at 24 °C (±0.5). For this, the insects were placed in 10 mL-metabolic chamber that received CO_2_-scrubbed air at a constant rate of 70 mL min^−1^ and connected to a sensor of CO_2_ (LI-6400; Li-Cor, Lincoln, USA). The mass-specific SMR was obtained averaging the 2-mins continuous and most stable VCO_2_ values (from the 10-mins register) and dividing this averaged value by the insect group weight. Aphids were discarded after the SMR measurements.

### Statistical analyses

The effects of the plant symbiotic status and SA application on the concentrations of SA and JA hormones, and on the plant above-ground biomass were analysed separately with linear effects models, using the function gls from the nlme package in R software (R Core Team, 2013), and assuming independent, identically distributed normal random errors (Pinheiro et al., 2009). The models included the plant’s symbiotic status (E+, E-) and SA treatment (SA+, SA-) as categorical factors. VarIdent variance structures were used on the SA treatments to accommodate deviations in the variance homogeneity in the SA and JA concentrations response variables (Zuur et al., 2009). After this procedure, all the ANOVA assumptions were met.

Similarly, the effects of the SA treatment on the concentrations of alkaloids (total lolines, NFL, and NANL) were analysed separately with lineal effect models, using the same software package mentioned earlier, and assuming independent, identically distributed normal random errors. The models included the SA treatment (SA+, SA-) as a categorical factor. When required, we used the function VarIdent on the SA treatment to accommodate deviations in the variance homogeneity (Zuur et al., 2009). ANOVA assumptions were then met.

The effects of plant symbiotic status and SA treatment on the population size of aphids (number of individuals) were analysed with linear mixed-effects models using the package glmmADMB in R software, and assuming that random errors were distributed independently and following a negative binomial distribution (Fournier et al., 2012). The model included plant symbiotic status (E+, E-), SA treatment (SA+, SA-), and time (7d and 14d since the aphid challenge) as categorical factors, and the random effect included the time nested in pot. Temporal autocorrelation between the repeated measurements was not observed.

The effects of the plant symbiotic status and the SA treatment on mass-specific SMR of aphids were analysed with linear effects models using the same R package and assuming the same random error distribution that for hormone concentration variables. The model included the plant symbiotic status (E+, E-) and SA treatment (SA+, SA-) as categorical factors. All the ANOVA assumptions were met. We performed post-hoc analyses between treatments when significant interactions were detected using the package *lsmeans* in R (Lenth, 2016). All the presented values in the result section are means ± standard errors (S.E.M). All data obtained in the present study are available in Table S1.

## Results

### Effects of plant endophyte presence and SA on hormone levels and plant growth

**Figure 1:**
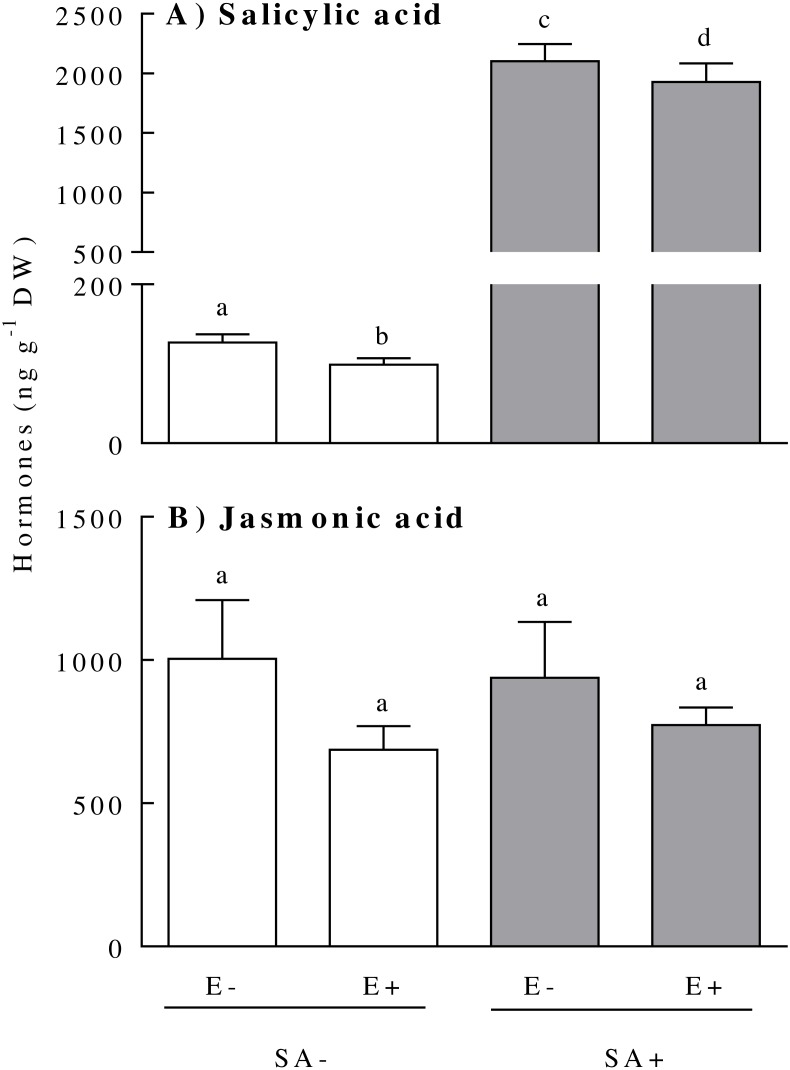
Physiological concentration of salicylic acid (panel A) and jasmonic acid (panel B) of *Lolium multiflorum* plants symbiotic with the endophyte fungus *Epichloë occultans*. Concentrations were measured three days after the salicylic acid application [treated: SA+ (shaded bars), and untreated: SA− (unshaded bars)] on *L. multiflorum* plants with (E+) and without (E−) the endophyte fungus. Different letters indicate significant differences at *P* < 0.05. Bars represent mean values ± S.E.M. (*n* = 8).

The concentration of the hormones SA and JA within the plants responded differentially to the endophyte presence and the exogenous application of the SA. The plant SA concentration was independently affected by the plant endophyte status and the hormonal treatment (Table 1). The presence of the endophyte in the plant reduced the SA by *ca*. 10% [E-: 1114.00 ± 264.50, and E+: 1014.00 ± 248.00 (ng SA g^−1^ DW)]. In addition, both plant biotypes (i.e., endophyte-symbiotic and non-symbiotic) showed increased SA concentrations of about 19-fold 3 days after the exogenous application of the hormone (Fig. 1A). Although the interaction effect between the treatments was not significant (Table 1), the reduction in SA concentration due to the endophyte presence was much more evident in plants not exposed to SA (the mean SA concentration difference between E+ and E- plants in the SA- treatment was around 22%) (Fig. 1A). The concentration of JA was not significantly modified by either the plant endophyte status or by the SA treatment (Fig. 1B) (Table 1).

**Table 1:** Effects of plant symbiotic status (E+, E−) and the exposure to the hormone salicylic acid (SA+, SA−) on different response variables of *Lolium multiflorum* plants symbiotic with the endophyte fungus *Epichloë occultans*. Note that for lolines, only the effect of SA is evaluated since endophyte-free plants do not contain these alkaloids. NFL and NANL mean N-formylloline and N-acetylnorloline alkaloids, respectively. Statistically significant effects are highlighted in bold. Mean values, S.E.M, and post hoc statistical differences are shown in Figures 1 and 2.

Response variable	Treatment	*df*	*F*	*P*-value
Salicylic acid (ng g^−1^ DW) (*n* = 8)				
	Symbiosis	1,28	4.70	**0.038**
	SA	1,28	316.87	**<0.001**
	Symbiosis × SA	1,28	0.45	0.506
Jasmonic acid (ng g^−1^ DW) (*n* = 8)				
	Symbiosis	1,28	2.57	0.120
	SA	1,28	0.49	0.487
	Symbiosis × SA	1,28	0.25	0.614
Above-ground plant biomass (g) (*n* = 14)				
	Symbiosis	1,108	4.41	**0.038**
	SA	1,108	1.08	0.300
	Symbiosis × SA	1,108	3.86	0.052
Lolines (µg g^−1^ DW) (*n* = 8)				
Total	SA	1,14	0.25	0.626
NFL	SA	1,14	0.36	0.555
NANL	SA	1,14	0.06	0.809

The above-ground plant tissues at the end of the aphid challenge (day 27 from the application of SA) was 11% higher in endophyte-symbiotic than in non-symbiotic plants (E-: 4.42 ± 0.28 g, and E+: 4.99 ± 0.39 g). This effect was, however, not affected by the treatment with SA (Table 1).

### Effects of SA on fungal loline concentrations

**Figure 2:**
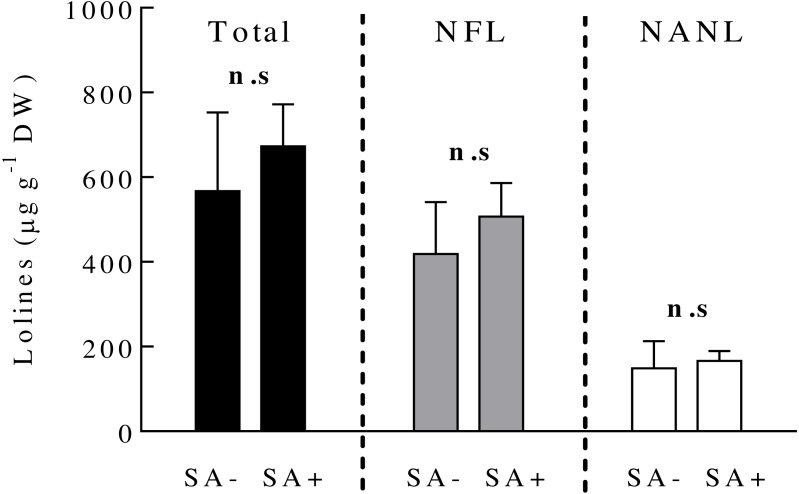
Concentrations of loline alkaloids produced by the fungal endophyte *Epichloë occultans* in *Lolium multiflorum* plants. Loline alkaloids were measured ten days after plants were exposed to salicylic acid (treated: SA+, untreated: SA−). Total lolines (black bars) are the sum of N-formylloline (NFL, grey bars) and N-acetylnorloline (NANL, white bars) derivatives. Non-symbiotic plants do not produce loline alkaloids. Each loline compound was analysed separately (see ‘Material and Method’ section). n.s. means non-significant differences between treatments. The bars represent mean values ± S.E.M. (*n* = 8).

Ten days after plant exposure to the SA hormone, the concentration of loline alkaloids (total and the derivatives NFL and NANL) in endophyte-symbiotic plants did not vary among SA-treated and SA-untreated plants (Fig. 2) (Table 1).

### Effects of plant endophyte presence and SA on *S. maydis* populations

**Figure 3:**
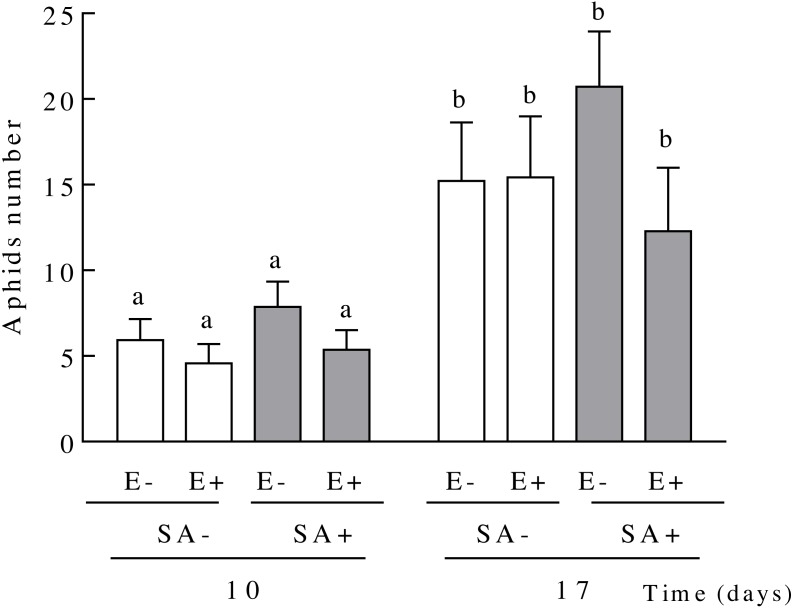
Population sizes of *Sipha maydis* aphids grown on *Lolium multiflorum* plants exposed to the salicylic acid hormone and symbiotic with the endophyte fungus *Epichloë occultans*. Aphids number were measured at days 10 and 17 post salicylic acid application [treated: SA+ (shaded bars), and untreated: SA− (unshaded bars)] on *L. multiflorum* plants with (E+) and without (E−) the endophyte fungus. Different letters indicate significant differences at *P* < 0.05. The bars represent mean values ± S.E.M. (*n* = 14).

The aphid population size on *L. multiflorum* plants increased over time but this increase was independent of both the endophyte presence or the plant exposure to salicylic acid (Table 2 and Fig. 3). On average, the aphid population size increased 2.60 fold in 7 days (from days 10 to 17 since plants exposure to SA, 5.96 ± 0.63 and 15.91 ± 1.74, respectively) (Fig. 3).

**Table 2:** Effects of plant symbiotic status (E+, E−) and the exposure salicylic acid (SA+, SA-) on aphids number and standard metabolic rate of *Sipha maydis* aphids grown on *Lolium multiflorum* plants with the endophyte fungus *Epichloë occultans*. Aphids number were measured at days 10 and 17 post salicylic acid application. Specific statistical differences for ‘aphids number’ response variable are shown in Figure 3. The volume of CO_2_ produced by aphids is abbreviated as ‘VCO_2_’. No significant differences were observed in standard metabolic rate (SMR) values. Replicate numbers are indicated in parenthesis. Values are mean ± S.E.M.

Response variable	Treatment	*df*	*χ*^2^ or F	*P*-value	SA−		SA+	
					E−	E+	E−	E+
Aphids number (*n* = 14)								
	Symbiosis	1,52	2.30	0.127	–	–	–	–
	SA	1,52	0.01	0.922	–	–	–	–
	Time	1,52	7.26	**0.007**	–	–	–	–
	Symbiosis × SA	1,52	0.32	0.567	–	–	–	–
	Symbiosis × Time	1,52	0.76	0.382	–	–	–	–
	SA× Time	1,52	1.52	0.217	–	–	–	–
	Symbiosis × SA× Time	1,52	1.37	0.241	–	–	–	–
Aphid mass-specific SMR (µL VCO_2_ h^−1^ mg^−1^) (*n* = 5)								
	Symbiosis	1,16	0.28	0.601	5.53 ± 0.91	5.13 ± 0.56	4.98 ± 0.89	4.38 ± 0.59
	SA	1,16	0.52	0.479				
	Symbiosis × SA	1,16	0.07	0.794				

### Effects of plant endophyte presence and SA on *S. maydis* metabolic rate

The aphid mass-specific SMR, evaluated at day 24 since the insects were placed on the plants, was unaffected by the endophytic fungus, the SA hormone, or the interaction between them (Table 2).

## Discussion

Since *Epichloë* fungi produce anti-herbivore alkaloids (Schardl et al., 2013a; Schardl et al., 2013b), we expected that endophytes would provide protection to host plants against the aphid *S. maydis*. However, we found that neither populations nor individuals of this aphid species were affected by the endophyte presence in plants. Despite the fact that aphids are usually controlled by the SA-dependent defence pathway (Thaler, Humphrey & Whiteman, 2012), here *S. maydis* aphids were not sensitive to the plant hormone exposure. Consistent with previous reports involving the plant interaction with beneficial microorganisms, the concentration of SA was lower in presence of the *Epichloë* endophyte fungus (Bastías et al., 2018a; Bastías et al., 2018b). However, the concentrations of alkaloids produced by endophytes was not affected by the plant exposure to SA.

The protection that each endophyte alkaloid type confers to host plants depends on, among other factors, the herbivore species (Bastias et al., 2017a; Bastías et al., 2017b). For example, loline fungal alkaloids confer effective protection against *Rhopalosiphum padi* aphids (Wilkinson et al., 2000), but the same alkaloids were ineffective in controlling *Heteronychus arator* beetles (Ball, Miles & Prestidge, 1997). In the present study, the presence of *E. occultans*, a loline producing endophyte fungus, did not confer protection to host *L. multiflorum* plants against *S. maydis* aphids. Similar findings were obtained in previous experiments studying the growth of *S. maydis* populations on the same plant-endophyte species system under field and laboratory conditions (Chaneton & Omacini, 2007; Miranda, Marina & Chaneton, 2011). In meadow fescue grass (*Festuca pratensis*) the presence of *E. uncinatum*, well-known to produce high concentrations of loline alkaloids, did not affect the population growth of *S. maydis* (Sabzalian, Hatami & Mirlohi, 2004). In that same study, however, the endophyte *E. coenophiala* (formerly *Neotyphodium coenophialum*) in tall fescue (*Schedonorus arundinacea*; formerly *F. arundinacea*) did effectively control *S. maydis* populations (Sabzalian, Hatami & Mirlohi, 2004). The difference between these two grass-endophyte symbioses, in terms of alkaloid profiles, is that while *E. uncinatum* only produces lolines, *E. coenophiala* synthesises ergopeptine and peramine alkaloids (Schardl et al., 2013a; Schardl et al., 2013b). Peramine and ergopeptine alkaloids produced by *Epichloë* endophytes are known for producing bioactivity against insects (Rowan, Hunt & Gaynor, 1986; Fleetwood et al., 2008). Thus, it is possible that the inefficiency of *E. occultans* endophytes in controlling *S. maydis* aphids in *L. multiflorum* is due, in part, to the particular profile of alkaloids produced by this endophyte species (Siegel et al., 1990).

The SA-dependent defence pathway is usually involved in plant responses to aphid attacks (Thaler, Humphrey & Whiteman, 2012; Ballaré, 2014). In the present study, the aphid *S. maydis* was not affected by the induction of the SA pathway (triggered by the plant exposure to the hormone). This insect insensitivity to plant SA-dependent defences has been reported for other aphids species (Bastías et al., 2018a; Bastías et al., 2018b; Onkokesung et al., 2016; Selig et al., 2016). The *S. maydis* insensitivity to SA-dependent defences could be explained by the potential capability of this insect species in detoxify *L. multiflorum* anti-herbivore metabolites. Detoxification of plant toxins is performed by enzymes that can deactivate or neutralize these metabolites (Després, David & Gallet, 2007). The synthesis of these detoxification enzymes is however, generally costly for insects, and these costs can be captured by metabolic measurements (e.g., Castañeda et al., 2010). In our study, however, *S. maydis* aphids grown on SA-treated *L. multiflorum* plants did not show any changes in their standard metabolic rates. To our knowledge, efficient mechanisms of detoxification of plant’s toxins have not been described for *S. maydis* aphids, which would be consistent with our findings for SMR. Adjustments in the feeding behaviour is another strategy that *S. maydis* aphids could have used to cope with plant defences (Walling, 2008). For instance, the aphid *Sitobion avenae* can reduce the time spent sucking phloem when feeding on SA-treated *Triticum aestivum* plants (Cao, Wang & Liu, 2014). In addition, it is also possible that an effective defence against *S. maydis* aphids in *L. multiflorum* requires more complex responses than just SA induction. For example, it has been documented that defence responses to aphids in *Arabidopsis thaliana*, *Glycine max*, and *Sorghum bicolor* plant species involves several hormone pathways that are sequentially induced during attacks by aphids (e.g., JA, SA, ethylene) (Moran & Thompson, 2001; Zhu-Salzman et al., 2004; De Vos et al., 2005; Li et al., 2008), and more recently that non-hormonal pathways can also be involved in these plant responses (i.e., methyl-D-erythritol-4-phosphate pathway) (Onkokesung et al., 2019).

Despite the fact that the fungal endophyte did not increase the resistance level of host plants against *S. maydis* aphids, endophyte-symbiotic plants may have been more tolerant to the aphid feeding due to the higher biomass they produced compared to their non-symbiotic counterparts. This growth promotion of *L. multiflorum* plants in presence of fungal endophytes has also been documented in other studies (Vila-Aiub, Gundel & Ghersa, 2005; Ueno et al., 2015; Bastías et al., 2018b). Since we had no aphid-free treatments, we cannot discard the possibility that the growth promotion documented in the present study had been a plant response to the aphid feeding more than a response to the endophyte presence (or a combination of both). However, findings from other studies suggest that this growth promotion might be a plant response to the endophyte presence. For example, another study using a similar experimental set-up to that used in the present work showed that endophyte-symbiotic *L. multiflorum* plants had a higher biomass than endophyte-free plants, and that this growth enhance was independent of the herbivory by *R. padi* aphids (Ueno et al., 2015).

It has been proposed that beneficial plant symbionts may regulate the SA pathway of the host to facilitate their own growth within plant tissues (Pozo & Azcón-Aguilar, 2007; Bastias et al., 2017a; Bastías et al., 2017b). This hypothesis has emerged from studies showing that the SA pathway can regulate the growth of these symbionts (Khaosaad et al., 2007; López-Ráez et al., 2010). In support of this, we observed that the plant SA concentration was reduced in presence of *Epichloë* fungal endophytes (see also Bastías et al., 2018a). In addition to regulating the growth of symbionts within plant tissues, the SA pathway can also modulate the functioning of beneficial symbiotic microorganisms (Hayat et al., 2010; Bastías et al., 2018a; De Vries et al., 2018). For example, the nitrogen fixation gene *NifE* in the beneficial cyanobiont, *Nostoc azollae*, was downregulated when *Azolla filiculoides* host plants were treated exogenously with methyl salicylate (De Vries et al., 2018). Based on these previous studies, we expected that symbiotic plants exposed to SA would show reduced levels of fungal alkaloids compared to untreated plants. Nevertheless, 10-days after the plants were exposed to the hormone, the concentration of loline alkaloids was similar between SA-treated and SA-untreated symbiotic plants. This result does not support our previous work which showed that alkaloid concentrations of endophyte-symbiotic plants were indeed reduced by SA treatment (Bastías et al., 2018a). It may be the case that in the present study, after an initial drop caused by the plant exposure to SA, fungal alkaloids were able to recover to their pre-treatment concentrations. Rapid increases in fungal alkaloids concentrations have been previously reported in plants symbiotic with *Epichloë* fungal endophytes. For example, we previously found that alkaloid concentrations took less than 7-days to respond to aphid herbivory (Bastías et al., 2018a).

## Conclusions

The present study indicates that the aphid *Sipha maydis* is insensitive to the anti-herbivore defences of *L. multiflorum* in symbiosis with *Epichloë occultans*. Our results indicate that neither the presence of *E. occultans* endophytes nor the induction of plant SA pathway regulate *S. maydis* populations. However, endophyte-symbiotic plants may have been more tolerant to the aphid feeding because these plants produced more aboveground biomass. We suggest that this insect insensitivity could be explained by a combination between the ineffectiveness of loline alkaloids (produced by *E. occultans*) in controlling *S. maydis* aphids and the capacity of this herbivore to tolerate the hormone-dependent defences of *L. multiflorum*.

##  Supplemental Information

10.7717/peerj.8257/supp-1 Table S1 Raw data Plant: Lolium multiflorum; fungal endophyte: Epichloë occultans; aphid: Sipha maydis.

## Additional Information and Declarations

### Data Availability

The following information was supplied regarding data availability: The raw data are available on figshare: Bastías, Daniel A; Martínez-Ghersa, Maria Alejandra; Newman, Jonathan A; Card, Stuart D.; Mace, Wade J.; Gundel, Pedro E. (2019): Table S1_Data.xlsx. figshare. Dataset. https://doi.org/10.6084/m9.figshare.9345524.v1.
